# Friends in Need

**Published:** 1892-09-10

**Authors:** 


### Passing Topics.

### FRIENDS IN NEED.

No better proof could be offered of the position occupied by the voluntary hospitals than the reliance instinctively placed upon them by the public as soon as cholera threatened to become epidemic in the metropolis. With one accord the whole community, and what is more to the purpose, the community's official representatives, turned to them in the emergency and, as was only to be expected, the hospitals responded with alacrity to the demand. Each has vied with the rest in placing its resources at disposal, and not a little of the equanimity with which the prospect, undoubtedly grave at one time, has been regarded, is due to the fact that the voluntary hospitals to the utmost of their ability have seconded the efforts of the Governmental departments, and by the timely provision of accommodation, not otherwise attainable, have helped to avert all occasion for panic.

Thus, not by any means for the first time in their history, the hospitals have placed society under weighty obligation and have proved themselves friends indeed. To people who take the trouble to think, hospital service, no doubt, appears infinitely greater in its continuous dealings with everyday sickness and accident than in meeting the passing demands of an extraordinary emergency, but human nature, as we know, is apt to despise all that is familiar, and to award a more or less exaggerated importance to whatever is uncommon. An invasion of these islands by cholera has not yet been frequent enough to inspire contempt, and what we hope we may now describe as the recent scare ought to attract to our struggling institutions a kindly attention which for a long time past has been conspicuously wanting.

If our voluntary hospitals are admittedly in the first line of defence both against native ills and the incursion of epidemic from abroad, it is not favourable to our reputation as a practical people, to say nothing of our philanthropic pretensions, that these important organisations should lack the funds necessary to their efficiency. This reflection appears to be in the mind of the Chairman of the Poplar Hospital, who in his letter to the *Times* makes forcible protest against the apathy which prevents his institution from compassing the strictly reasonable and praiseworthy end it has in view. The Poplar Hospital is a minor but most useful charity. It stands in the thick of the danger-fraught turmoil of the great port of London, and concerns itself only with such cases of emergency, accident and others, which cannot safely bear transport to the nearest general hospital, distant two miles. If cholera should be anywhere in the metropolis, in all likelihood it would be found in full force at Poplar, and the chances of cholera-stricken sufferers are not improved by a long journey. It is within the modest programme of the authorities to take a share in the treatment of local cases, but for want of money they can only provide two beds ! Well may the Chairman show a pardonable impatience. We only want "a wretched £3,000 to enable us to complete our rebuilding," he cries. Ah ! Mr. Sydney Holland, fellow feeling makes us entirely in sympathy with you, and not a bit sarcastic when we say—we wish you may get it.

Agricola.

